# On the Rational Design of Cooperative Receptors

**DOI:** 10.1146/annurev-biophys-091222-082247

**Published:** 2023-02-03

**Authors:** Gabriel Ortega, Alejandro Chamorro-Garcia, Francesco Ricci, Kevin W. Plaxco

**Affiliations:** 1 Precision Medicine and Metabolism Laboratory, CIC bioGUNE, Derio, Spain; 2 Ikerbasque, Basque Foundation for Science, Bilbao, Spain; 3 Department of Chemistry and Biochemistry, University of California, Santa Barbara, California, USA; 4 Biological Engineering Graduate Program, University of California, Santa Barbara, California, USA; 5 Dipartimento di Scienze e Tecnologie Chimiche, University of Rome Tor Vergata, Rome, Italy

**Keywords:** allosterism, aptamers, binding-induced folding, bioengineering, biosensors, population shift, synthetic biology

## Abstract

Cooperativity (homotropic allostery) is the primary mechanism by which evolution steepens the binding curves of biomolecular receptors to produce more responsive input–output behavior in biomolecular systems. Motivated by the ubiquity with which nature employs this effect, over the past 15 years we, together with other groups, have engineered this mechanism into several otherwise noncooperative receptors. These efforts largely aimed to improve the utility of such receptors in artificial biotechnologies, such as synthetic biology and biosensors, but they have also provided the first quantitative, experimental tests of longstanding ideas about the mechanisms underlying cooperativity. In this article, we review the literature on the design of this effect, paying particular attention to the design strategies involved, the extent to which each can be rationally applied to (and optimized for) new receptors, and what each teaches us about the origins and optimization of this important phenomenon.

## INTRODUCTION

The complexity of biological systems creates a need for mechanisms by which nature can tune the responsiveness of biomolecules to small changes in the concentration of their target ligands. Hemoglobin (26), for example, must pick up oxygen in the lungs and deliver it to the tissues over a small (typically only approximately threefold) and often varying (with altitude or exercise) concentration gradient. One of the most widely used approaches by which nature modulates the binding behavior of enzymes, transporters, and receptors is allostery (30), a mechanism in which the binding of one ligand alters the affinity with which subsequent ligands bind. This effect was first reported by Christian Bohr and colleagues (7), who, in 1904, discovered that carbon dioxide and protons decrease hemoglobin’s oxygen affinity. The term allostery, from the Greek roots allo (other) and stereo (solid, three-dimensional), however, was not coined until 1961, when Jacques Monod & François Jacob (35) used the phrase to describe the mediation of ligand binding at one site on a receptor by the binding of a ligand or ligands to other, nonoverlapping binding sites (for a particularly readable history, see 9).

**Figure 1:**
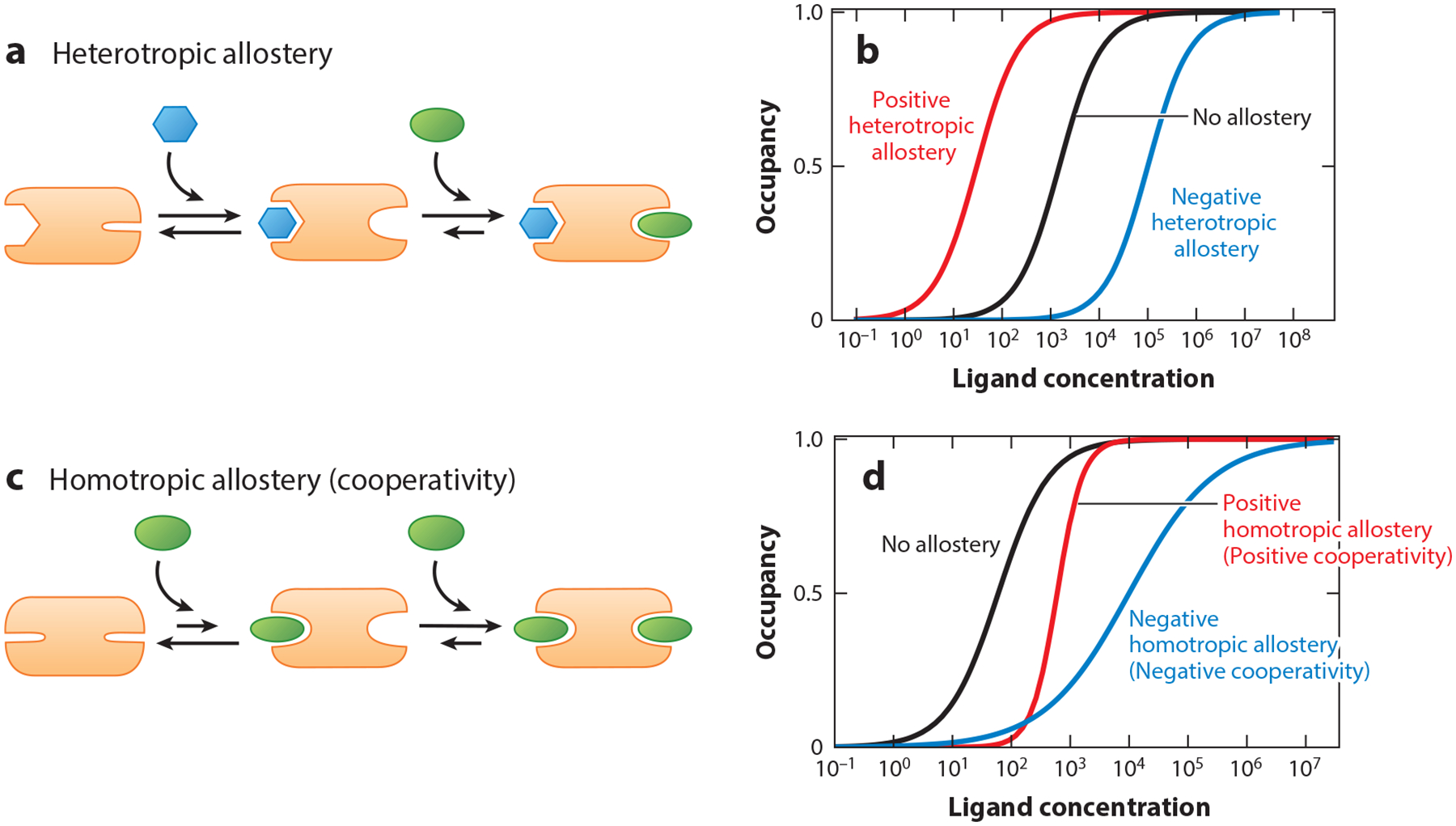
Nature uses allostery to tune the shape and position of binding curves. (*a,b*) In heterotropic allostery, the binding of one ligand to the receptor increases (*red* in panel *b*) or decreases (*blue* in panel *b*) the affinity with which another, different ligand binds. This shifts the binding curve toward lower or higher ligand concentrations without altering its shape. (*c,d*) In homotropic allostery, which is also known as cooperativity, the binding curve is steepened. In negative cooperativity, in contrast, the binding curve is flattened. Homotropic allostery thus renders the system more (positive cooperativity) or less (negative cooperativity) sensitive to small changes in ligand concentration.

The two (or more) ligands involved in allostery can be either distinct molecules, an effect termed heterotopic allostery, or two or more copies of the same molecule, which is termed homotropic allostery but is also known as cooperativity. In the former, the binding of one ligand either increases (positive allostery) or decreases (negative allostery) the affinity with which the second, different molecule binds (Figure 1*a*), shifting the binding curve toward lower or higher ligand concentrations without changing its shape (Figure 1*b*). In the latter, the binding of one copy of a ligand modulates the affinity with which additional copies of the same ligand bind (Figure 1*c*). This type of allostery changes both the placement and the shape of the binding curve, producing either a steeper (i.e., more responsive), higher-order dependence on ligand concentration (positive cooperativity) or a shallower, lower-order dependence (negative cooperativity) (Figure 1*d*). Like heterotropic allostery, cooperativity was first identified based on early 1900s observations of hemoglobin, which was found to bind oxygen with an approximately third-order (i.e., third-power) dependence on oxygen partial pressure (4, 7, 20). This results in a steeper, more responsive binding curve, which enhances the protein’s ability to pick up and then deliver oxygen efficiently over the rather modest concentration gradient present between the lungs and peripheral tissues. Over the century since that observation, cooperativity has been found to occur in a wide range of biological processes, including the regulation of metabolism (5, 6, 18, 23, 29, 39), the transport of ions (34) and neurotransmitters (17), and the regulation of gene transcription and translation (12, 24, 42). It is this mechanism, and rational efforts to introduce it into otherwise noncooperative receptors, that is the focus of our review.

**Figure 2:**
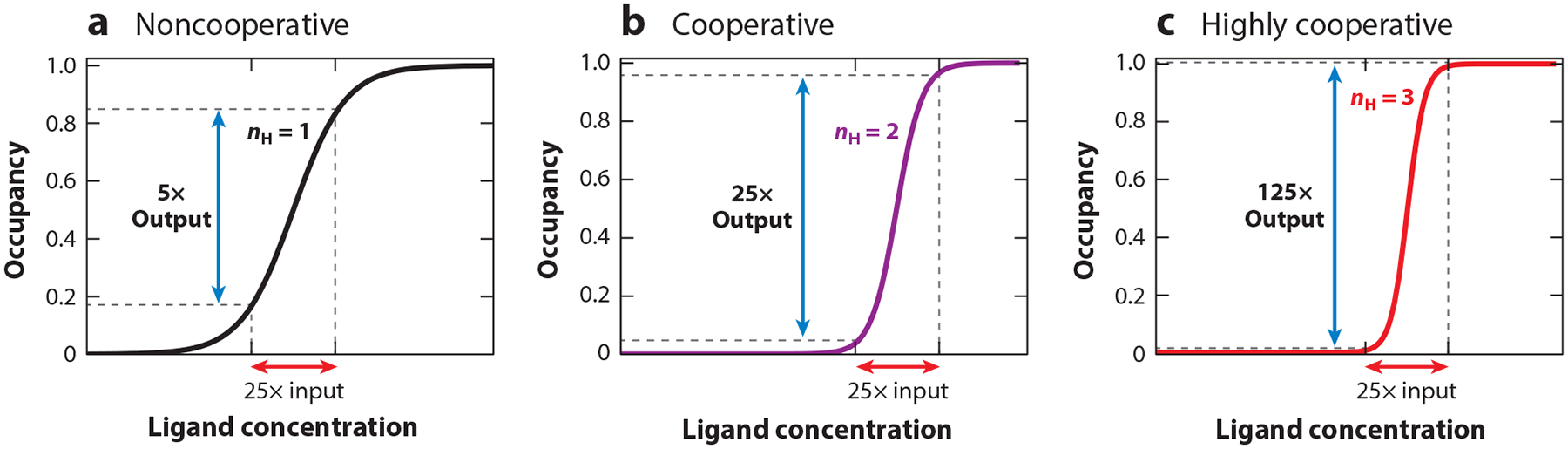
To illustrate the power of cooperativity, we present the change in occupancy produced by a 25-fold change in ligand concentration (from fivefold below to fivefold above the binding midpoint) on the occupancy of receptors exhibiting different degrees of cooperativity. For example, (*a*) whereas for a noncooperative receptor, this leads to only a fivefold change in occupancy, (*b*) for a modestly cooperative receptor (i.e., of Hill coefficient 2), the input and output reach parity. (*c*) At still greater cooperativity (i.e., a Hill coefficient of 3), the output is amplified by fivefold relative to the input. Throughout this review, we employ semilog plots for clarity; we note that this causes the hyperbolic shape of the noncooperative binding curve (panel *a*) to appear as a sigmoid.

The occupancy of noncooperative receptors follows a hyperbolic dependence on target concentration. By altering the shape of this binding curve, cooperativity renders a receptor’s occupancy more (positive cooperativity) or less (negative cooperativity) sensitive to small changes in ligand concentration (16, 58). For example, for a noncooperative receptor, a 25-fold change in ligand concentration (from fivefold below the binding midpoint to fivefold above) produces only a fivefold change in receptor occupancy and any resulting output (Figure 2*a*). In contrast, when the Hill coefficient (a measure of cooperativity that we discuss in detail below) reaches 2, the same 25-fold change in input (ligand concentration) leads to an equivalent 25-fold change in output (Figure 2*b*). By the time the Hill coefficient reaches 3, this 25-fold change in input produces a 125-fold change in output, amplifying the signal by a factor of 5 (Figure 2*c*).

The ubiquity of cooperativity in biology suggests that it may also be of value in artificial biotechnologies, where it can be used to enhance the responsiveness of the receptors used in biosensors or synthetic pathways. For example, biosensors that respond more dramatically to small changes in the concentration of their target ligand enable more precise measurements. This is useful in applications seeking a binary yes/no response, such as the detection of pregnancy or the diagnosis of infection (8, 33, 45, 55), or when measuring drugs characterized by very narrow therapeutic windows, for which small differences in target concentration mark the difference between the desired effect and toxicity (3, 27, 51, 52). Motivated by such examples, over the past 15 years researchers have increasingly been engineering cooperativity into otherwise noncooperative receptors. These same efforts have also provided the first experimental tests of longstanding ideas about the mechanisms underlying cooperativity.

## THE BINDING CURVES OF COOPERATIVE AND NONCOOPERATIVE RECEPTORS

Most biological recognition elements present only a single binding site. The binding of such receptors follows the hyperbolic relationship between the input (ligand concentration) and the output (receptor occupancy) that, despite having been described by A.V. Hill (19) a few years earlier (for a readable history, see 11), is called the Langmuir Isotherm (25):

1. $$\theta =\frac{\left[L\right]}{[L]+K_{d}}.$$

In this equation, the fraction of receptors occupied, θ, is determined by the concentration of the target ligand, [L], and the receptor’s affinity for that ligand, $K_{d}$, which is the target concentration at which half of the receptors are occupied. The useful dynamic range (which the field arbitrarily defines as the change in ligand concentration required to transition occupancy from 10% to 90%) associated with such binding is 81-fold, which is so broad that the sensitivity of such receptors to small changes in target concentration is poor. This relative insensitivity to small changes in ligand concentration has been amusingly termed the “tyranny of the Langmuir isotherm” (13).

Cooperativity employs multiple, interacting binding sites to alter the steepness of binding curves, thus narrowing or broadening their useful dynamic range and increasing or decreasing the sensitivity with which receptor occupancy changes with changing ligand concentration. This occurs when the first ligand to bind a multisite receptor increases (positive cooperativity) or decreases (negative cooperativity) the affinity with which subsequent copies of the same molecule bind. This leads to, respectively, higher- or lower-order dependencies of occupancy, θ, on ligand concentration, [L], an observation that was first described empirically by A.V. Hill (20) in 1910 in a single-author paper that he published while still an undergraduate:

2. $$\theta =\frac{[L{]}^{n_{H}}}{[L{]}^{n_{H}}+{K_{1/2}}^{n_{H}}}.$$

Because the multiple sites on a cooperative receptor differ in affinity, we replace the $K_{d}$ of individual binding sites with an overall dissociation constant, $K_{1/2}$, denoting the concentration of ligand at which half of a cooperative receptor’s sites are occupied. The exponent introduced in this equation, the Hill coefficient, $n_{H}$, is a measure of the degree of cooperativity. Noncooperative receptors exhibit a Hill coefficient of 1. Positively cooperative systems, in contrast, exhibit a higher-order dependence on ligand concentration and a Hill coefficient greater than 1, with the Hill coefficient asymptotically approaching its maximum value (defined by the number of binding sites) as the degree of cooperativity increases. Conversely, Hill coefficients below 1 produce negative cooperativity, with a lower (than unity)-order dependence on ligand concentration.

**Figure 3:**
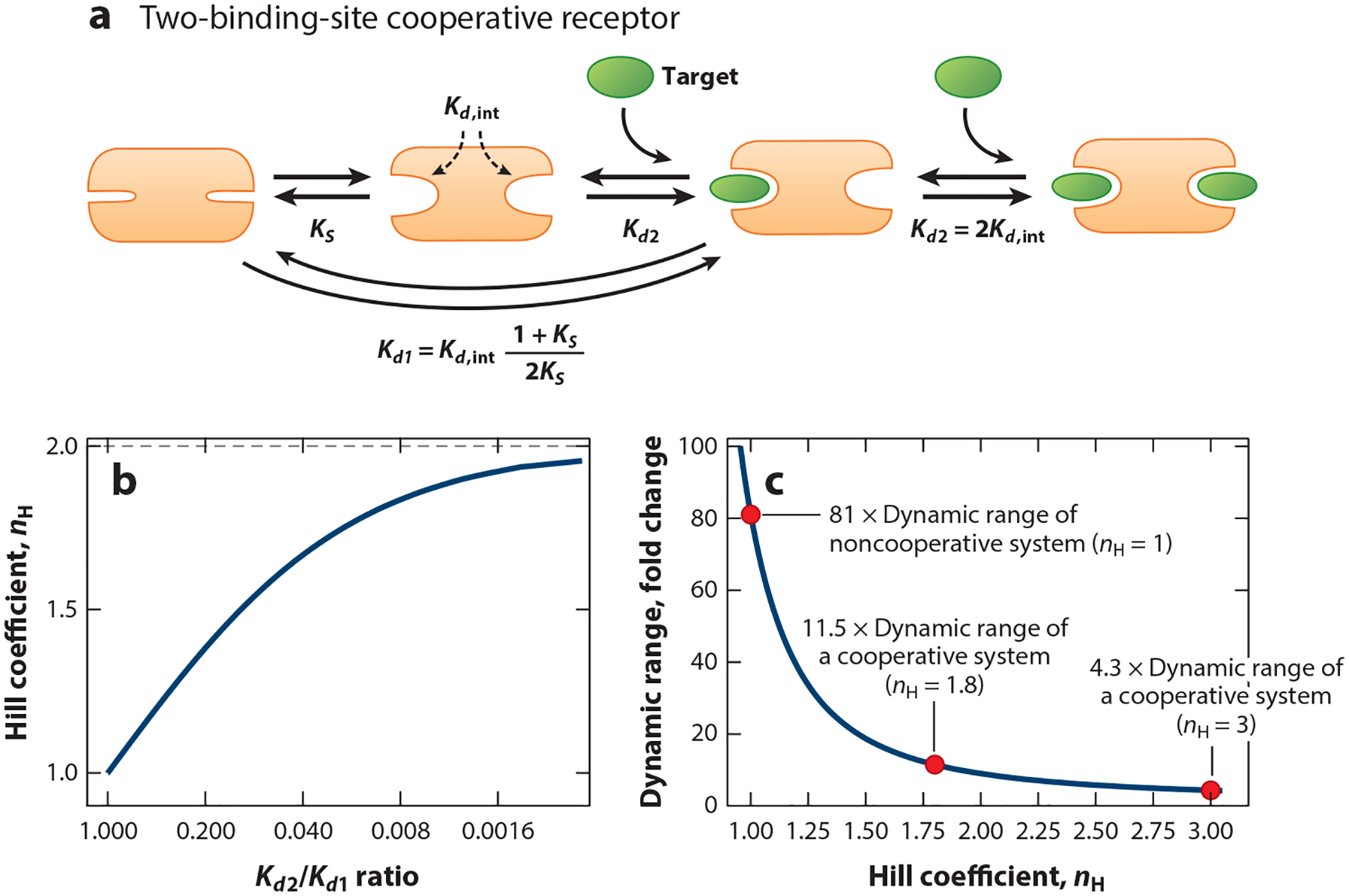
The relative affinities of the binding events on a multisite receptor determine its degree of cooperativity. (*a*) Most often, the necessary binding energy landscape is achieved by coupling the first binding event to an unfavorable conformational change (denoted by the equilibrium constant, $K_{S}$), reducing its affinity, $K_{d1}$, relative to the intrinsic affinity, $K_{d,\text{int}}$, of a properly formed binding site. The second binding event, in contrast, does not pay this energetic cost (since it takes place on a properly formed binding site), enhancing its affinity, $K_{d2}$, and generating cooperativity. Note the factors of 2 in $K_{d1}$ and one-half in $K_{d2}$, corresponding to statistical corrections arising due to the two binding sites involved (46). (*b*) Shown is the extent to which the Hill coefficient varies for a two-site receptor as a function of the ratio of the affinities of the two binding events (Equation 4). Note that the Hill coefficient asymptotically approaches 2 as the ratio of the binding affinities goes to 0 (i.e., as the affinity of the second binding event becomes infinitely greater than that of the first). (*c*) Fortunately, however, the dynamic range (defined in this review as the relative concentration change required to transition the receptor from 0.1 to 0.9 occupancy) is a fairly strong function of the Hill coefficient (Equation 3), leading to near ideal cooperativity at Hill coefficients well below the limiting case.

The higher-order dependence on ligand concentration produced by positive cooperativity steepens a receptor’s binding curve (Figure 3*c*; Table 1), with the relationship between the Hill coefficient and the resulting signal amplification (the relative change in occupancy for a given relative change in ligand concentration) being given by

3. $$\text{relative}\;\text{change}\;\text{in}\;\text{occupancy}=(\text{relative}\;\text{change}\;\text{in}\;\text{ligand}\;\text{concentration}{)}^{\frac{n_{H}}{2}}.$$

This renders cooperative receptors more responsive to small changes in target concentration. As shown in Figure 2, for example, whereas a 25-fold change in ligand concentration around the binding midpoint of a noncooperative receptor only changes its occupancy by fivefold, for a Hill coefficient of 2 the change in occupancy rises to 25-fold, and for a Hill coefficient of 3 it rises to 125-fold (Table 1). With this, a given uncertainty in the measurement of a receptor’s output translates into a lower uncertainty in the estimated ligand concentration when cooperative receptors are employed in biosensor applications. For example, whereas, for a noncooperative receptor, an uncertainty of 1% in the measurement of the receptor occupancy translates into an uncertainty of 4% in ligand concentration, this resulting uncertainty decreases to only 2% and 1% for receptors with Hill coefficients of 2 and 4, respectively (Table 1).

## THE THERMODYNAMICS OF COOPERATIVITY

To generate the binding energy landscape required to produce cooperativity, most cooperative systems couple the first binding event to an unfavorable structural rearrangement that, in turn, increases the affinity of the remaining binding sites. Typically, this occurs via a population shift mechanism, in which, in the absence of a target, the receptor is in equilibrium between a more stable configuration whose biding sites are of low (or, in the case of many examples of designed cooperativity, effectively no) affinity and a less stable conformation whose binding sites are higher in affinity (32, 47, 48, 54, 57) (Figure 3*a*). The first binding event then pays the cost associated with the unfavorable conformational change to the higher affinity state, reducing affinity. Subsequent binding events, in contrast, need not pay this energetic cost, and thus their affinity will be higher than that of the first binding event. In this mechanism, the individual binding sites can all be of the same affinity (e.g., all the binding sites in Figure 3*a* exhibit the same intrinsic affinity, $K_{d,\text{int}}$). We note, however, that the binding sites in a cooperative system can be heterogeneous. Under these circumstances, differences in individual affinities can mask positive cooperativity, producing lower Hill coefficients or even apparent negative cooperativity. Such systems have seen little if any rational design and thus fall beyond the scope of this review; we refer the reader to more specialized studies (e.g., 44).

Receptors asymptotically approach perfect cooperativity (i.e., a Hill coefficient exactly equal to the number of binding sites) as the affinity of subsequent binding events becomes ever higher than that of the first. Conveniently for biomolecular engineers, however, this asymptotic curve rapidly converges on near-ideal behavior. For a two-site receptor, for example, the relationship between cooperativity and the ratio of the affinities for the first and second binding events, $K_{d1}$ and $K_{d2}$, is given by

4. $$n_{H}=\frac{2}{1+\sqrt{\frac{K_{d2}}{K_{d1}}}},$$

or, equivalently, by the difference in the binding free energies, $\Delta G_{B1}$ and $\Delta G_{B2}$, of the individual binding events

5. $$n_{H}=\frac{2}{1+e^{-\frac{\Delta G_{B1}-\Delta G_{B2}}{2\text{RT}}}}.$$

Given these relationships, a Hill coefficient of 1.8, which reduces the width of the useful dynamic range from 81-fold to just 11-fold (versus the ninefold that would be seen for ideal cooperativity), is reached when the affinity of the second binding event is increased just 81-fold, corresponding to only a 10.9 kJ/mol change in binding free energy at 298 K (Figure 3*b*,*c*).

The benefits of cooperative binding notwithstanding, the effect comes at a cost: Steeper binding curves are associated with a reduction in overall affinity. Specifically, the overall binding energy, $\Delta G_{b}$, of a cooperative receptor is given by the arithmetic mean of the binding free energies of each individual binding event, $\Delta G_{b}(i)$:

6. $$\Delta G_{b}=\frac{\sum_{i=1}^{n}\Delta G_{b}(i)}{n}.$$

Because of this, the binding midpoint of a cooperative receptor, $K_{1/2}$, is given by the geometric mean of the affinities of the individual binding events, $K_{d}(i)$:

7. $$K_{1/2}={\left[\prod_{i=1}^{n}K_{d}(i)\right]}^{1/n},$$

where n is the number of binding sites. As noted above, however, near-ideal cooperativity requires only relatively small changes in the affinity of the first and second binding events, and thus the affinity cost associated with cooperativity need not be prohibitive. As discussed in detail below, this cost can be at least partially overcome by generating receptors with more binding sides.

## ENGINEERING COOPERATIVITY

The past 15 years have seen slow but steady progress regarding the rational introduction of cooperativity into normally noncooperative receptors, which we review in this section. While these efforts have primarily been driven by the practical interest in improving the sensitivity (to small changes in ligand concentration) of the receptors used in artificial biosystems, such as biosensors (53, 56, 57) or synthetic biological pathways (14), work in this field has also provided an opportunity to dissect the thermodynamics of cooperativity in what we believe to be informative, insight-generating detail. We note, too, that all of the work to date regarding the rational design of cooperativity has focused on the design of positive cooperativity. This is because increased sensitivity is more often of utility in biotechnologies, where it can be used, for example, to enhance measurement precision, than is decreased sensitivity. That said, decreased sensitivity does have the benefit of broadening the dynamic range. However, for those rare technological problems that require a dynamic range broader than the 81-fold afforded by simple Langmuir isotherm binding, this broader range is easily achieved using sets of noninteracting receptors varying in affinity, which obviates the need to introduce allostery (22, 43).

**Figure 4:**
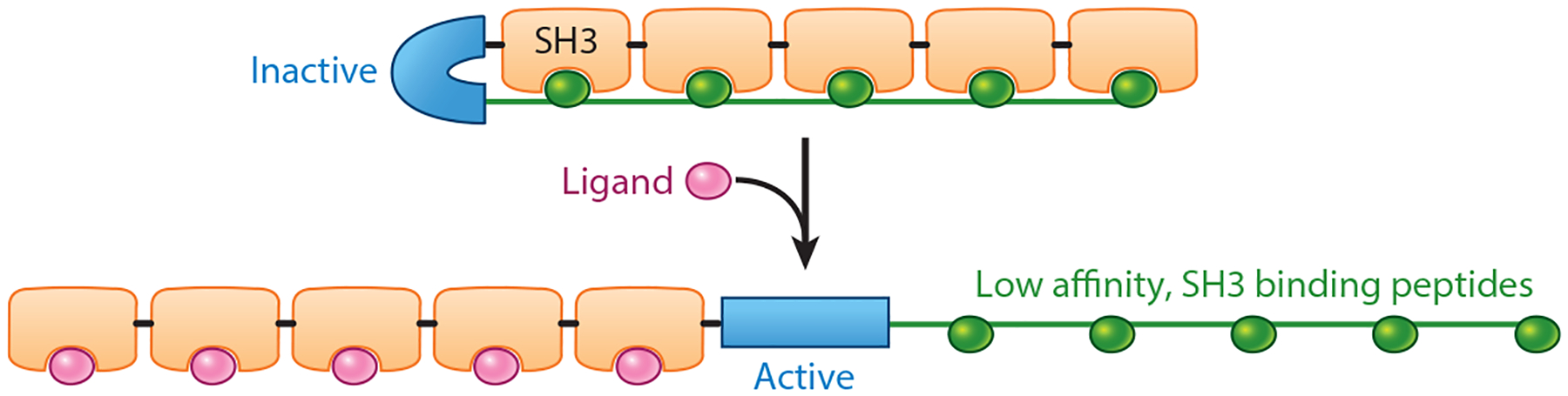
Dueber et al. (14) introduced up to (as illustrated) five FynSH3 domains on one side of a catalytic domain (used to produce a measurable output reporting on binding) and, on the other, an equal number of copies of a polypeptide that binds this domain. (*Top*) Upon binding to one another, these produce a conformational stress on the catalytic domain, thus inactivating it. (*Bottom*) Upon the addition of a peptide ligand that binds the SH3 domain with higher affinity, the stress is released, activating the catalytic domain in a cooperative fashion that achieves a Hill coefficient of ~3.9.

The first example that we are aware of in which positive cooperativity was introduced into a normally noncooperative receptor was the work of Dueber et al. (14), who, in 2007, described the design of a modular system employing a peptide-binding SH3 domain as the receptor. To render this receptor cooperative, they appended up to five copies of it to one side of the enzyme N-WASP and then attached up to five copies of a low-affinity, SH3-binding peptide on the other side (Figure 4). When these bind one another, the resulting steric strain distorts the structure of the enzyme, rendering it inactive. When the receptor’s target is added (a higher-affinity peptide ligand), it competes with this intermolecular binding, liberating the SH3 domains, releasing the strain, and activating the enzyme. As expected, a construct containing only a single SH3 domain and a single copy of the peptide target is noncooperative, with a Hill coefficient of ~1 (in this review, we use ~ to denote a lack of reported confidence intervals). Upon the introduction of five SH3 domains (and, correspondingly, five peptide recognition elements), the authors achieved a Hill coefficient of ~3.9 (Table 2 presents a summary of this and all of the other systems that we discuss).

**Figure 5:**
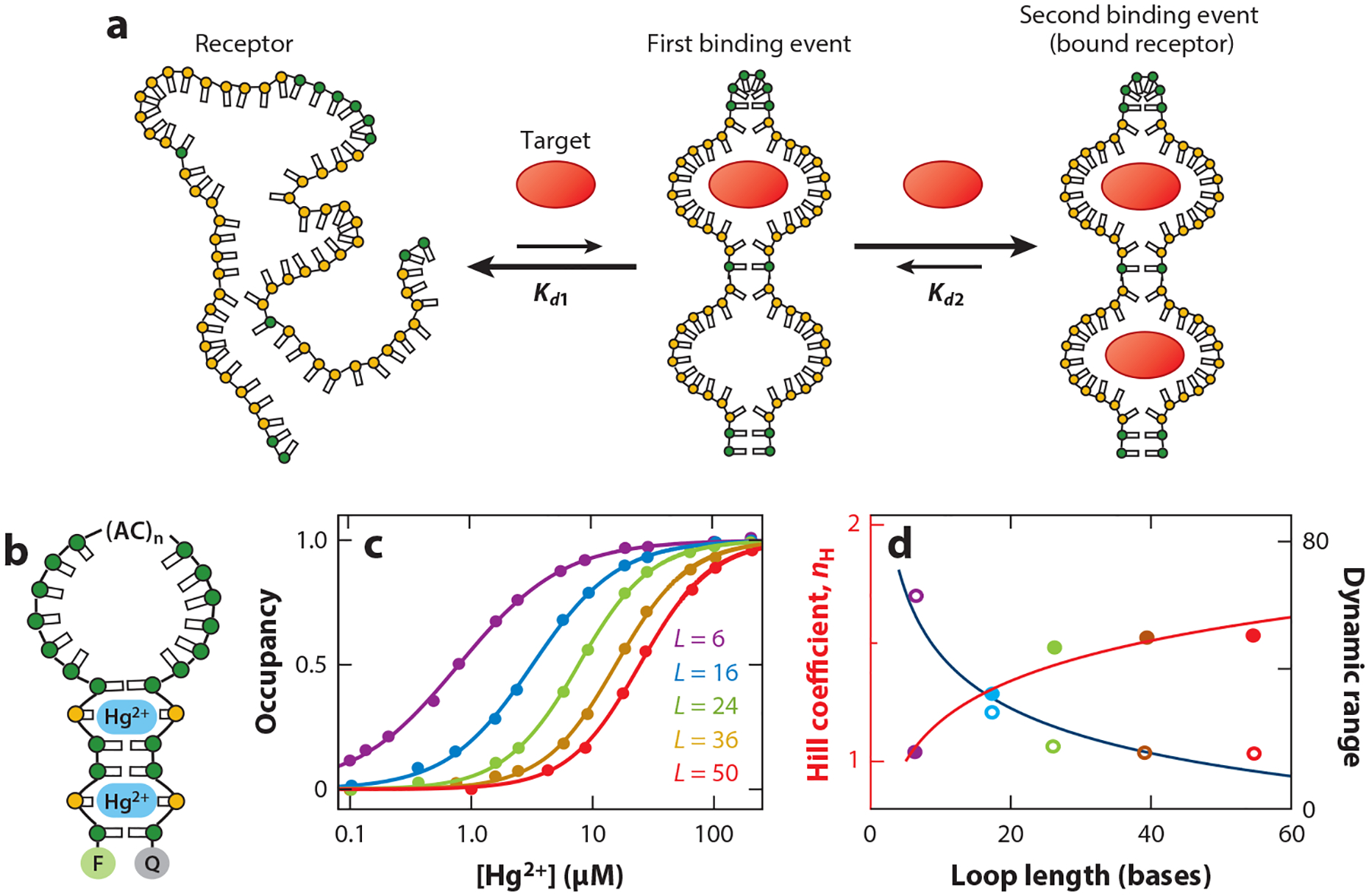
(*a*) Perhaps the most successful method of introducing cooperativity into normally noncooperative receptors has been to exploit the binding-induced folding of intrinsically disordered biopolymers. In this case, a biopolymer is designed that equilibrates between a (more stable) unfolded state, which does not bind the target, and a (less stable) folded state capable of binding two or more copies of the target. (*b*) We have explored this mechanism quantitatively using a short DNA helix containing two mercury-binding thymine–thymine mismatches (see 47). The large entropy associated with closing the loop to form the ligand-binding stem renders the system unfolded in the absence of a target, generating the required binding-induced folding. The fluorophore (F) and quencher (Q) pair situated on the termini in these constructs (and those shown in Figures 6–9) provides a convenient optical output. (*c*) By increasing the length of the connecting loop, the energetic cost of forming the double-stranded stem is increased, thus increasing cooperativity. (*d*) The Hill coefficients (and, correspondingly, dynamic ranges) of the resulting receptors scale as expected given the logarithmic dependence of loop closure energy on loop length and the known relationship (Equation 4) between cooperativity and the relative affinities of the first and second binding events (*solid lines*). Panels *c* and *d* adapted from Reference 47.

The binding-induced strain approach that Dueber et al. (14) employed to create a cooperative receptor may suffer from poor generalizability. This is because it requires that (*a*) multiple copies of the ligand be attached to the receptor, which is likely not always easy to achieve, and (*b*) that the binding of the receptor to its intramolecular ligands causes sufficient strain (i.e., produces a large enough difference in energy between the binding events) to produce a cooperative output, which likely depends sensitively on the details of the attachment geometry. Presumably because of these complexities, this strain-based approach to generating cooperativity has seen no follow up. Instead, all of the more recent successes in rationally introducing cooperativity into otherwise noncooperative receptors have employed a different mechanism, binding-induced folding (also known as intrinsic disorder), as a means of coupling target recognition to the necessary conformational change (Figure 5*a*).

In the first example employing binding-induced folding as a means of generating cooperativity, Wang et al. (53) designed a synthetic mercury-binding DNA sequence consisting of a DNA strand that is unfolded in the absence of its target. In the presence of mercury, however, it folds into a hairpin structure comprised of a four-base loop and a double helix containing seven mercury-binding thymine–thymine mismatches. As the stem is unstable in the absence of mercury, the first ligand to bind must pay the unfavorable energy associated with closing the loop and forming the double helix. Subsequent binding events, in contrast, occur on a preformed double helix, enhancing their affinity and producing a Hill coefficient of ~2.4.

Building on the binding-induced folding approach, several authors have reported cooperative oligonucleotide-based receptors binding a range of other ligands. In the first of these, Mullen et al. (37) linked together multiple unfolded, potassium-binding RNA G-quadruplexes to achieve Hill coefficients of up to 2.7 ± 0.1. We note, however, that the authors also observed Hill coefficients well above 1 for receptors that, in principle, bind only a single potassium ion. This suggests that some of the cooperativity is arising due to effects other than allosteric cooperativity. The increase in ionic strength associated with the addition of hundreds of millimolar potassium ions, for example, could mimic cooperativity by stabilizing the folded, target-binding conformation of the oligonucleotide independently of the stabilization caused by (specific) ligand binding events. In another example, our group has linked multiple melamine-binding thymine triplets in a single strand of DNA, achieving Hill coefficients of 2.7 ± 0.1 for a four-site construct and 2.9 ± 0.1 for a six-site construct (in work from our group, all confidence intervals reflect estimated 95% confidence levels) (28). In parallel, our group has also designed a series of multisite DNA nanodevices that bind a short DNA strand as their ligand (32). As its recognition elements, this receptor employs two identical copies of the same DNA sequence that form a 2:1 triple helix with a second, complementary DNA sequence (the ligand) via the formation of both Hoogstein and Watson-Crick-Franklin base pairing. To generate a cooperative receptor, we fabricated a series of hairpins containing one or more pairs of the recognition sequence connected by a flexible, 22-base linker, such that the first binding event again must pay the energetic cost of closing the loop. The resulting receptors achieve Hill coefficients ranging from 1.1 ± 0.1 for the noncooperative single-site construct to 2.4 ± 0.2 for a three-site construct.

The use of binding-induced folding to engineer cooperativity provides a readily achievable, rational approach to tuning the degree of cooperativity. Using a simple system structurally similar to the mercury-binding receptor of Wang et al. (53), our group has explored this question quantitatively. To do so we employed a stem-loop whose six-base-pair stem contains two thymine–thymine mismatches (see 47) (Figure 5*a*). Lengthening the construct’s loop enhances cooperativity by increasing the energetic difference between the first and second binding events. For example, extending the loop from 6 bases to 50 shifts the Hill coefficient from 1.05 ± 0.05 to 1.51 ± 0.03 (Figure 5*b*). Consistent with this, we see an excellent correlation between the cooperativity of these constructs and the known relationship (Equation 5) between the Hill coefficient and the energy gap between a two-site receptor’s first and second binding events, which follows the expected logarithmic dependence on loop length (see 10, 41) (Figure 5*c*). Nesterova and coworkers (38) have, similarly, used hairpin modifications to alter the folding thermodynamics of a DNA i-motif containing 10 proton binding sites, pushing the 5.3 ± 0.6 Hill coefficient of the parent construct to 8.1 ± 0.5 in their most cooperative construct.

**Figure 6:**
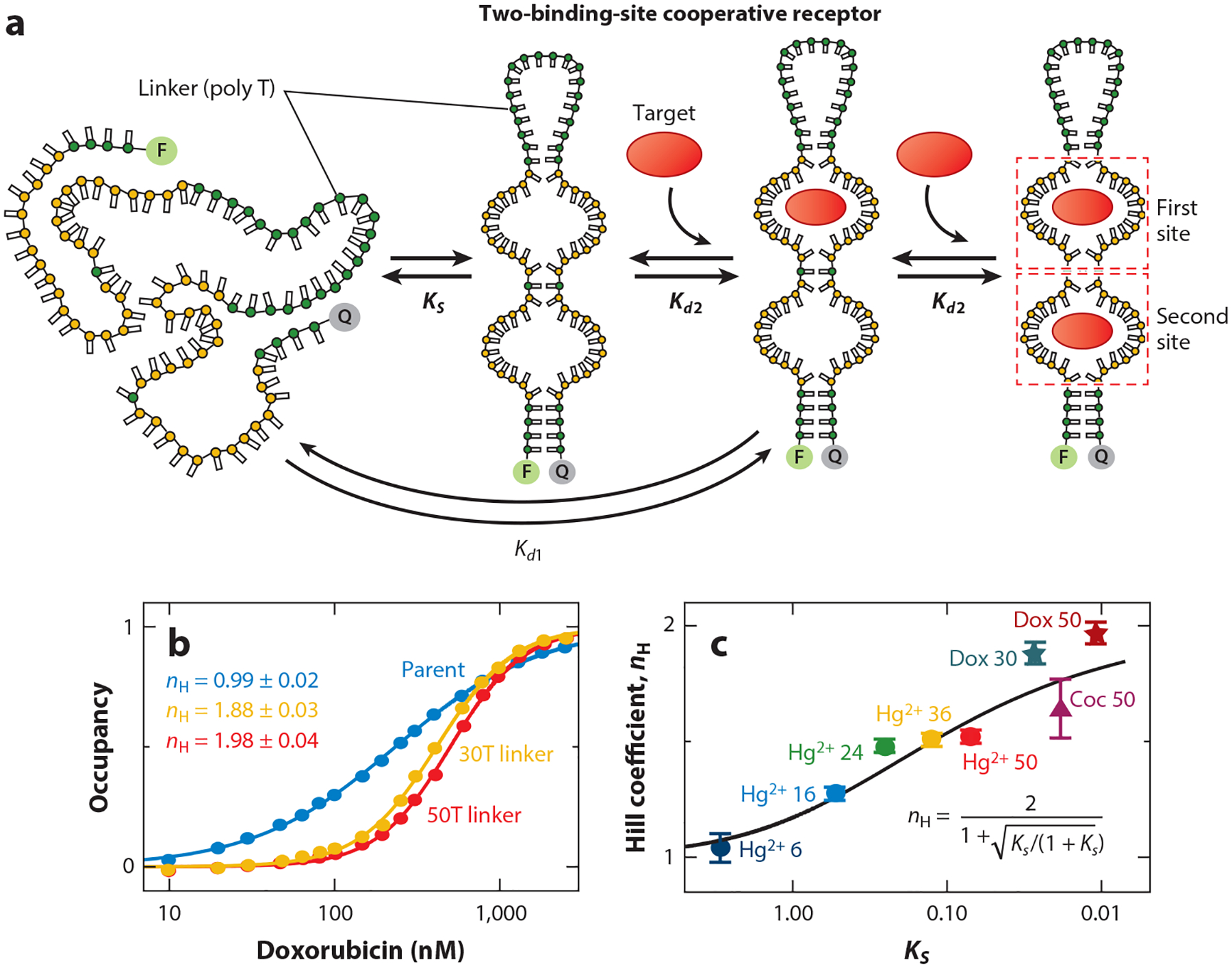
(*a*) The split-aptamer approach to engineering cooperativity. In this case, we generate constructs comprised of tandem repeats of one part of a receptor connected by a flexible linker to tandem repeats of the other portion of the receptor. As the first binding event must pay the thermodynamic cost associated with closing the linker to bring the split-aptamer pairs together, its affinity is reduced. The second binding event happens on a preformed binding site, increasing its affinity and leading to cooperativity. (*b*) Shown are binding curves for the parent (single-site) doxorubicin-binding aptamer and two cooperative, two-site constructs varying in the length of their linker and, thus, in the energetic gap between the two binding events. (*c*) As is the case with our mercury-binding receptors (also shown), the cooperativity of these split-aptamer constructs correlates well with the energetic cost associated with closing the loop to form the first binding site, with the latter being presented here in terms of the equilibrium constant, $K_{S}$, for this conformational switch. Panels *b* and *c* adapted from Reference 47.

The above examples of the rational introduction of cooperativity relied on fairly simple receptors, such as mercury-binding thymine–thymine mismatches, and potassium-binding G-quadruplexes. Some years ago, however, we demonstrated a method by which we can introduce cooperativity into structurally complex receptors. Indeed, using our approach, we have introduced cooperativity into several aptamers [DNA molecules selected in vitro to bind a specific molecular target (2, 15, 21, 49)] of unknown structure. To achieve this, we (somewhat arbitrarily) split the aptamer into two pieces; fabricated tandem repeats of each piece; and linked these together via a long, flexible poly-thymine loop to create a single, two-site receptor (see 47) (Figure 6*a*). In the absence of a target, the entropic cost of closing this loop keeps the receptor unfolded, reducing the affinity of the first, but not the second, binding event. Using this approach, we achieved a Hill coefficient of 1.65 ± 0.12 for a cocaine-binding aptamer and of up to 1.98 ± 0.04 for a doxorubicin-binding aptamer, with the latter being within the error of the maximum cooperativity attainable for a two-site receptor (Figure 6*b*). As was the case with our mercury-binding receptors, the cooperativities measured for these split-aptamer constructs correlate well with the equilibrium constants (i.e., the energetic costs) associated with closing their loops (Figure 6*c*).

**Figure 7:**
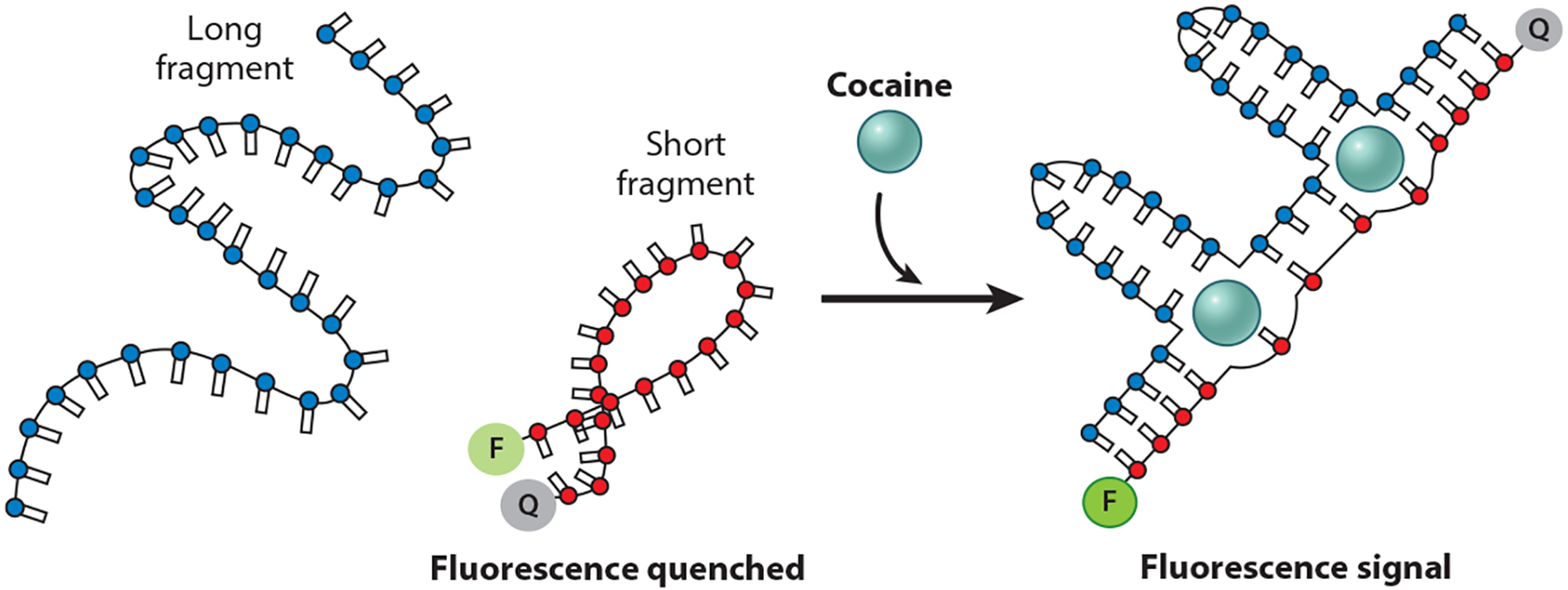
The Xiao group (57) has pioneered a bimolecular split-aptamer approach in which the tandem repeats of the two portions of the aptamer are on separate DNA strands, such that the binding of the first ligand must overcome an unfavorable biomolecular association. Using this approach, they have designed cooperative aptamers binding several ligands including, as shown, cocaine, for which the cooperative receptor achieved a Hill coefficient of ~1.5.

Building on our split-site strategy, Xiao and coworkers (57) have created cooperative systems that reduce the affinity of the first binding event by splitting a two-site receptor into two unconnected elements (Figure 7). This mechanism can be seen as a particular case of the use of disordered loops described above, in which the entropic penalty corresponds to that of a loop so long that the formation of the binding sites is controlled by the free diffusion of the two portions of the receptor. Using this approach, researchers have designed cooperative, two-site aptamers against cocaine, reaching a Hill coefficient of ~1.5 (57); against the cathinones (a large family of mostly illicit drugs), reaching a Hill coefficient of ~1.8 (31); and against dehydroisoandrosterone-3-sulfate (a naturally occurring steroid hormone precursor), reaching a Hill coefficient of ~1.6 (56).

While binding-induced folding provides a convenient route to the introduction of cooperativity, we have also demonstrated examples in which the receptor transitions between two conformations of well-defined structure. We have done so while introducing cooperativity into molecular beacons, which are simple, optically reporting receptors comprised of a single strand of DNA with self-complementary ends, such that it forms a stem-loop (see 50). The hybridization of a target oligonucleotide complementary to the single-stranded loop ruptures the double-stranded stem, segregating a fluorophore–quencher pair placed on the oligonucleotide’s termini and leading to enhanced emission. To date, we have demonstrated two molecular beacon architectures that introduce cooperativity into these otherwise noncooperative receptors.

**Figure 8:**
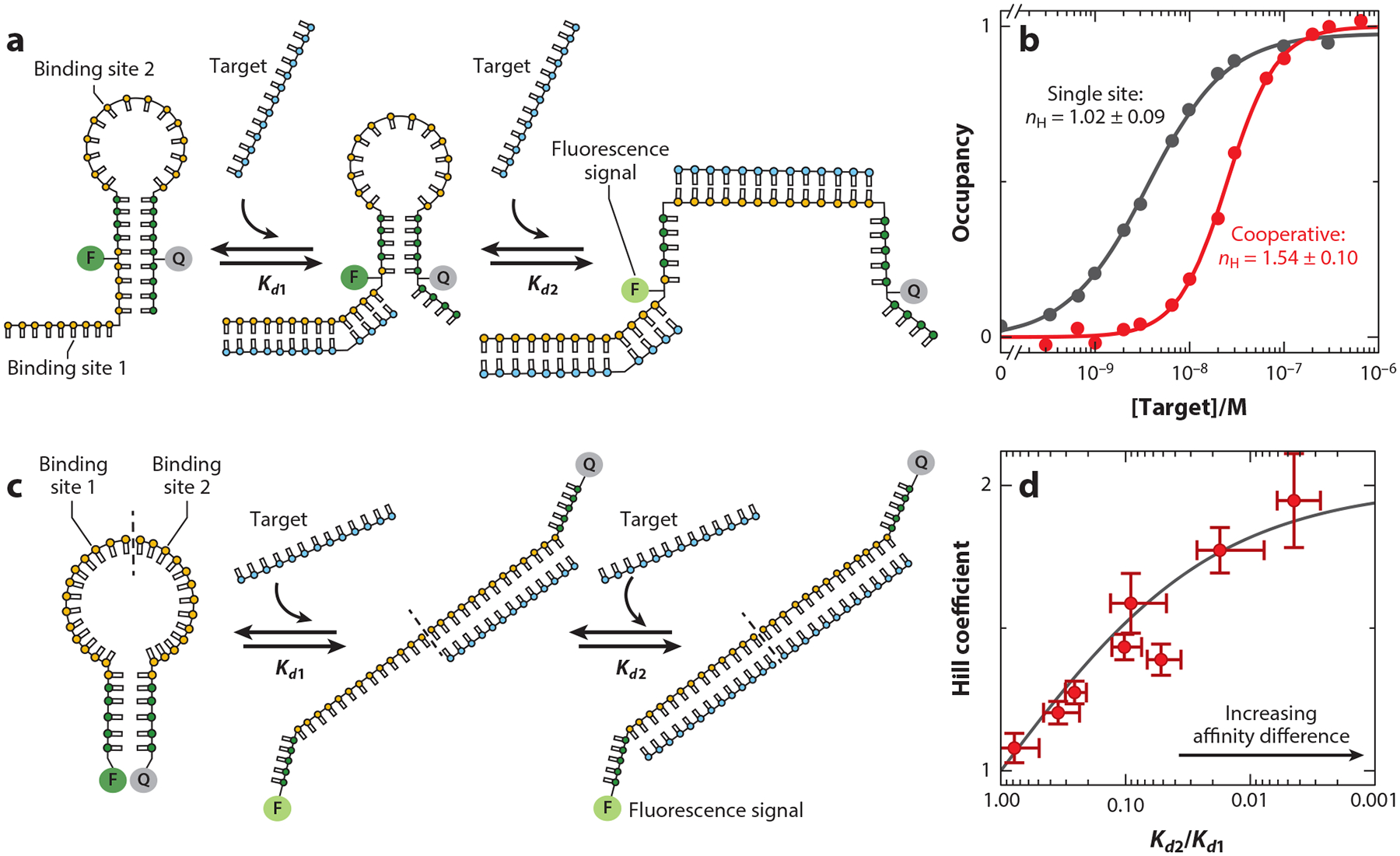
Our group has developed two approaches to introducing cooperativity into molecular beacons, which are stem-loop DNA constructs commonly employed as sensors for the detection of specific nucleic acids (see 50). (*a*) In the first of these, we added a second binding site contained partially within the 5’ strand of the stem and partially within an appended single-stranded tail. Binding to either the loop or the tail disrupts the stem, rendering the second binding event higher in affinity. (*b*) As expected, the binding of an unmodified molecular beacon is noncooperative, producing a Hill coefficient of 1.02 ± 0.09. The tailed beacon, in contrast, achieves a Hill coefficient of 1.54 ± 0.10. (*c*) Our second design places two target-binding sites within the beacon’s single-stranded loop, rendering it possible to stabilize the stem (i.e., increase the energy gap between the two binding events) without altering the receptor’s specificity. Using this architecture, we have engineered receptors with Hill coefficients within error of the ideal maximum of 2 (see 48). (*d*) Using control constructs (in which the two binding sites are nonequivalent) to determine $K_{d1}$ and $K_{d2}$, we have shown that the relationship between the Hill coefficient and the ratio of the affinities of the two binding events holds even as the latter varies over many orders of magnitude. Panels *b* and *d* adapted from Reference 48.

Our first cooperative molecular beacon architecture adds a single-stranded tail to the receptor, such that the beacon now contains two identical binding sites, one in the loop, which is blocked from binding via stress induced by the formation of the stem, and a second on the tail, which partially overlaps with the stem (Figure 8*a*). The binding of the first copy of the ligand to either of these sites opens the stem, exposing the second site, which can then bind a second copy of the ligand with higher affinity (see 48). The resulting affinity difference is sufficient to produce a Hill coefficient of 1.54 ± 0.10 (Figure 8*b*). Achieving higher Hill coefficients requires further increases in the energy gap between the first and subsequent binding events, which can be achieved by increasing the stability of the stem. In our first cooperative molecular beacon, however, the stem forms a portion of the ligand-binding site, such that altering it also affects the specificity and affinity of binding. In response, we next designed a cooperative architecture in which the stem is not involved in ligand recognition. Specifically, we introduced two identical binding sites within the molecular beacon’s loop, such that the enhanced stiffness of the first binding event opens the stem, increasing the affinity of the second binding event (Figure 8*c*). We then modulated the energy gap of the system by altering the GC content of its stem. Using this approach, we have tuned the Hill coefficient of such constructs from within error of unity to values as high as 1.94 ± 0.17. We have also used this system to explore the extent to which cooperativity varies with changes in the relative affinity of the two binding events. We have done so by changing the sequence of one of the binding sites (rendering the receptor heterotropically allosteric), which provides a means of measuring the affinity of the second binding event as a function of the occupancy of the first binding site. The degree of cooperativity seen for the eight constructs that we characterized using this approach closely follows the expected relationship between cooperativity and the relative affinities of the two binding events (Figure 8*d*).

## OPTIMIZING THE ENERGY LANDSCAPE OF COOPERATIVITY

The improved sensitivity associated with cooperativity comes at a price: It reduces a receptor’s affinity. That is, as cooperativity enhances the responsiveness of biomolecular receptors to small changes in the concentration of their target ligand, it concomitantly pushes the binding midpoint, which is given by the geometric mean affinity of all the binding sites (Equation 7), to higher ligand concentrations. The binding midpoint of a two-site receptor with a Hill coefficient of 1.9, for example, must be at least 19 times higher than the dissociation constant of the higher-affinity of its two binding sites.

**Figure 9:**
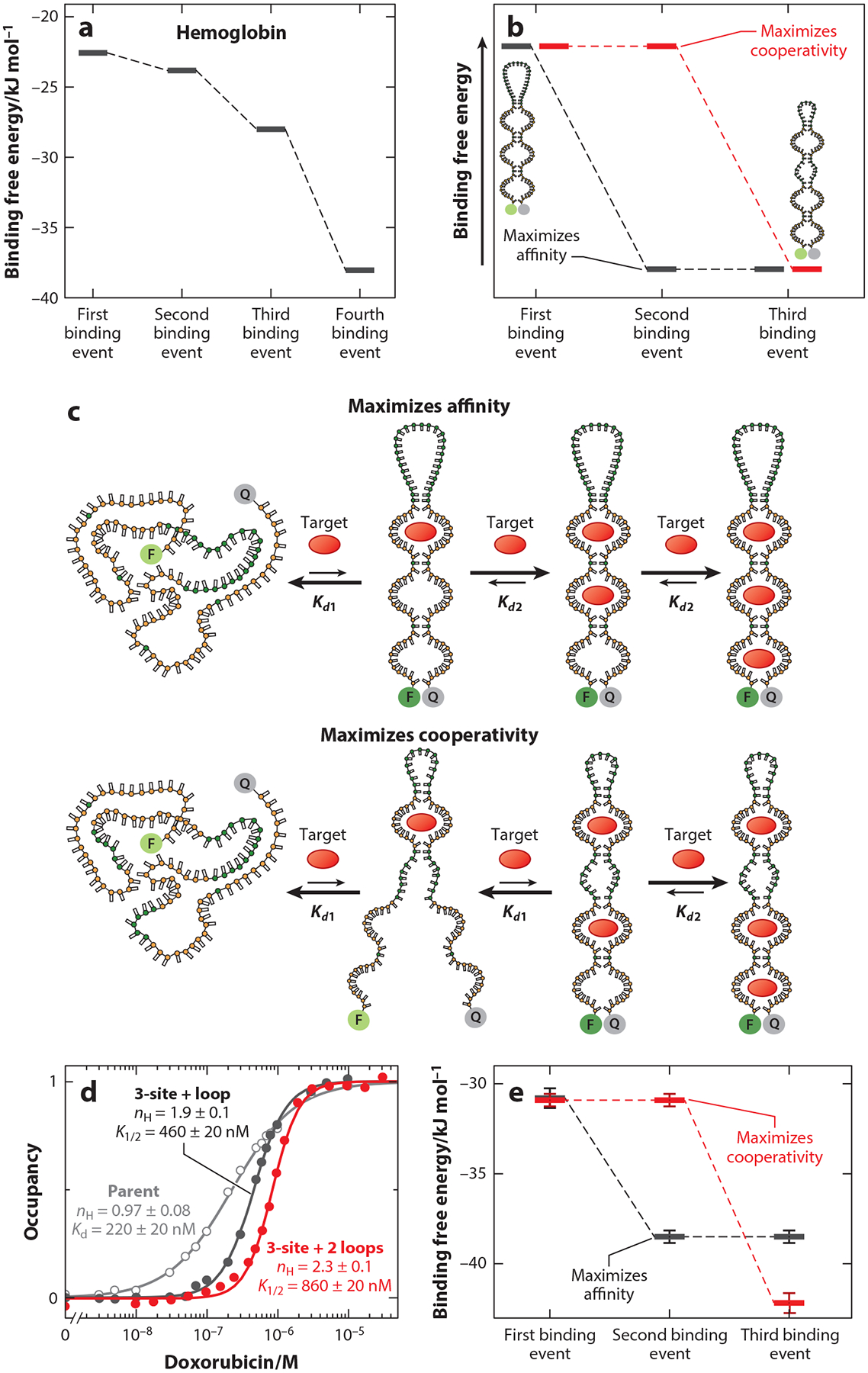
The addition of new binding sites enables optimizing the trade-off between cooperativity and affinity. (*a*) Hemoglobin, for example, uses this strategy, featuring three sites of lower affinity and a fourth of higher affinity to achieve Hill coefficients of ~3 while retaining high affinity. (*b*) Inspired by this, we rationalized that by using the intermediate binding sites as gateways, we could design binding energy landscapes maximizing affinity (*black trace*) or cooperativity (*red trace*). (c) To test experimentally these ideas, we generated a three-site receptor in which the first binding event pays all of the energetic cost of the conformational change (*top schematic*). Because the all-or-none binding event occurs as soon as the first ligand binds, the Hill coefficient is near 2, rather than near 3. However, because the second and third binding events on this construct are both high affinity, the overall binding affinity of this construct is high. To instead tune the binding energy landscape to favor cooperativity rather than higher affinity, we designed a construct in which the first two binding events are both similarly low in affinity (*bottom schematic*). This reduces the overall binding affinity, but it leads to all-or-none binding only after the second ligand binds and thus produces a higher Hill coefficient. (*d*) Consistent with our designs, the first construct (*black*) achieves a Hill coefficient of 1.9 ± 0.1 and a binding midpoint only ~twofold higher than that of the noncooperative parent receptor (*gray*). In contrast, the second construct (*red*) achieves a Hill coefficient of 2.3 ± 0.1 but at a cost of a binding midpoint ~fourfold higher than that of the parent receptor. (e) For our higher-affinity, lower-cooperativity construct (*black*), the first binding event is much less favorable than the second and third, which are quite close to that of the parent aptamer, thus reproducing the binding energy landscape predicted to be biased toward affinity rather than cooperativity. In contrast, the energy landscape of our second construct (*red*) features first and second binding events that are much less favorable than the third, biasing it toward cooperativity at the expense of affinity. Panels *d* and *e* adapted from Reference 40.

Increasing the number of binding sites in a cooperative receptor provides opportunities to tune the trade-off between affinity and cooperativity. Specifically, increasing the number of binding sites provides additional binding free energy, which can be used to further increase cooperativity, to enhance the affinity, or to perform some combination of the two. This strategy can also be found in nature, such as in the case of hemoglobin, which features four binding sites to achieve reasonably high cooperative binding while also retaining relatively high affinity. Specifically, upon dissecting the binding thermodynamics of hemoglobin, Gary Ackers et al. (1) found that the first three binding events occur at lower affinity than the fourth, thus creating a binding energy landscape that produces a Hill coefficient of ~3 while retaining a binding midpoint within a factor of ~14 of that of a high-affinity, noncooperative form of the protein (Figure 9*a*).

Exploring the above arguments via experimental design, we have recently created receptors in which we rationally partitioned the binding free energy to populate binding free energy landscapes that optimize either cooperativity or affinity (see 40). To do this, we employed the split-aptamer mechanism described above to design three-binding-site receptors starting from a single-site—and thus noncooperative—doxorubicin-binding aptamer. In our first effort, we designed a construct such that the affinity of the first, second, and third binding events are low, high, and high, respectively (Figure 9*b*,*c*). We did this by simply placing an additional split aptamer on the end of our previously described, two-site split aptamer doxorubicin receptor (see Figure 6). This maximizes the number of binding sites with high affinity, thus producing a binding energy landscape that partitions the energy provided by the additional binding event to favor higher affinity at the cost of lower cooperativity. Specifically, this construct achieves a Hill coefficient of 1.9 ± 0.1 but exhibits less than a twofold reduction in affinity relative to the parent, single-site aptamer (Figure 9*d*). In contrast, a two-site receptor of equivalent cooperativity would exhibit at least a 19-fold decrease in affinity (Equation 4). Alternatively, the energy associated with the additional binding events can be used to enhance cooperativity rather than affinity. To achieve this, we created a three-site receptor in which flexible loops connect not only the two parts of the first split aptamer, but also the two parts of the second split aptamer, producing affinities that are low, low, and high, respectively, at the three sites (Figure 9*b*,*c*). The resulting construct achieves a Hill coefficient of 2.3 ± 0.1 but with fourfold poorer affinity than that of the parent, single-site aptamer (Figure 9*d*).

Experimental dissection of the binding energy landscapes of the above-described three-site constructs confirms the mechanistic hypotheses underlying our design strategies. To perform this analysis, we extracted the affinities of each individual binding event by fitting our binding data to a Monod–Wyman–Changeux model of three-site cooperative binding (36, 40). This model assumes that only the fully formed sites bind the ligand (a generally accurate approximation for designed examples of cooperativity), and that the affinity of each site is modulated only by the energetic cost of closing its associated loops, and not by any differences in their intrinsic affinity. Using this model, we recovered estimated individual affinities that reproduce the energy landscape expected to produce improved affinity (Figure 9*e*), that is, a landscape in which the nearly all-or-none transition occurs after the first site is occupied, leading to an approximately second-order dependence on concentration (i.e., a Hill coefficient of near 2), and for which the average affinity of the three sites (and thus the overall affinity of the receptor) is high. In contrast, the binding energy landscape of the more cooperative three-site aptamer is such that, similarly to that of hemoglobin, the affinities of the first binding events are poorer than that of the last, such that the nearly all-or-none transition occurs only after the second site is occupied (Figure 9*e*). This leads to an approximately third-order dependence on concentration and a poorer average affinity.

## CONCLUSIONS

Recent years have seen slow but steady advances in our ability not only to introduce cooperativity into normally noncooperative receptors, but also to do so in a rational fashion that, increasingly, allows for the degree of cooperativity to be controlled. Using a binding-induced folding mechanism, for example, it has proven possible to introduce cooperativity into receptors of unknown structure and to tune the trade-off between affinity and cooperativity. In addition to providing quantitative, experimental tests of longstanding ideas about the thermodynamics underlying cooperativity, these efforts are paving the way toward rational improvement of the sensitivity of the biomolecules employed in artificial biotechnologies, such as synthetic biology and biosensors.

## Figures and Tables

**Table 1:** Dynamic range, occupancy change, and uncertainty in estimated ligand concentration

Hill Coefficient (*n*_H_)	Dynamic range (fold concentration change)	Fold change in occupancy for a 25-fold change in ligand concentration^a^	Uncertainty in estimated ligand concentration for a 1% uncertainty in occupancy^a^ (%)
1	81.0	5	4.0
2	9.0	25	2.0
3	4.3	125	1.3
4	3.0	625	1.0
5	2.4	3,125	0.8

a Centered on the binding midpoint.

**Table 2:** A summary of the efforts discussed in this review

Target	Type of receptor	Parent receptor			Approach	Engineered receptor			Reference
		*K*_D_ or *K*_1/2_	Number of sites	*n*_H_		*K*_1/2_	Number of sites	*n*_H_	
SH3 recognition peptide	SH3 peptide-binding domain	~0.1 μM	1	Not reported	Steric strain	~10 μM	5	3.9 ± 0.3	14
Mercury ions(Hg^2+^)	DNA (thymine-thymine mismatch)	NA	NA	NA	Binding-induced folding	~470 nM	7	~2.4	53
Potassium (K^+^)	RNA G-quadruplexes	0.8 ± 0.2 mM	1	1.7 ± 0.4	Binding-induced folding	117 ± 28 mM	1	2.7 ± 0.1	37
Melamine	Melamine-binding DNA sequence	490 ±10 μM	2	1.9 ± 0.1	Binding-induced folding	276 ±4 μM	6	2.9 ± 0.1	28
Short oligonucleotides	DNA triplex formation	560 ± 30 nM	1	1.1 ± 0.1	Binding-induced folding	~100 nM	2	2.4 ± 0.2	32
Mercury ions(Hg^2+^)	DNA(thymine-thymine mismatch)	~1 μM	2	1.05 ± 0.05	Binding-induced folding	~30 μM	2	1.51 ± 0.03	47
Protons	DNA i-motif	pH 7.00 ± 0.01	10	5.33 ± 0.57	Destabilization of binding-competent conformation	pH 7.04 ± 0.01	10	8.0 ± 0.5	38
Doxorubicin	DNA aptamer	~200 nM	1	0.99 ± 0.02	Split aptamer	~500 nM	2	1.98 ± 0.04	47
Cocaine	DNA aptamer	~100 μM	1	0.99 ± 0.02	Split aptamer	~3 mM	2	1.65 ± 0.12	47
Cocaine	DNA aptamer	~5 μM	1	~1	Bimolecular split aptamer	~36 μM	2	~1.5	57
Cathinones	DNA aptamer	~6 μM	1	Not reported	Bimolecular split aptamer	~140.6 μM	2	~1.8	31
Dehydroiso-androsterone-3-sulfate	DNA aptamer	264 ± 40 μM	1	1.05 ± 0.10	Bimolecular split aptamer	491 ± 24 μM	2	1.6 ± 0.1	56
Short oligonucleotides	Molecularbeacons	3.6 ± 0.4 nM	1	1.02 ± 0.09 0.98 ± 0.05	Switch between defined conformations	25 ± 2 nM notreported	2 2	1.54 ± 0.10 1.94 ± 0.17	48
Doxorubicin	DNA aptamer	220 ±20 nM	1	0.97 ± 0.08	Rational control of the binding energy landscape	460 ± 20 nM 860 ± 20 nM	3	1.9 ± 0.12.3 ± 0.1	40

Abbreviation: NA, not applicable.
